# Adverse fetal/neonatal and obstetric outcomes in pregnancies with both maternal and fetal heart disease

**DOI:** 10.1038/s41372-024-02058-3

**Published:** 2024-07-23

**Authors:** Beatriz A. Fernandez-Campos, Jasmine Grewal, Marla Kiess, Samuel C. Siu, Birgit Pfaller, Mathew Sermer, Jennifer Mason, Candice K. Silversides, Kim Haberer

**Affiliations:** 1 Division of Cardiology, University of Toronto, Pregnancy and Heart Disease Program, Mount Sinai and Toronto General Hospitals, Toronto, ON Canada; 2 Division of Cardiology, St Paul’s Hospital, University of British Columbia, Vancouver, BC Canada; 3 Division of Cardiology, University of Western Ontario, London, ON Canada; 4 Department of Internal Medicine 1, University Hospital of St. Pölten, Karl Landsteiner University of Health Sciences, Karl Landsteiner Institute for Nephrology, St. Pölten, Austria; 5 Division of Maternal Fetal Medicine, University of Toronto, Special Pregnancy Program, Mount Sinai Hospital, Toronto, ON Canada; 6 Division of Pediatric Cardiology, Cohen Children’s Medical Center of New York- Donald and Barbara Zucker School of Medicine at Hofstra/Northwell, New York, NY USA

**Keywords:** Congenital heart defects, Pre-eclampsia

## Abstract

**Objective:**

To investigate fetal/neonatal and obstetric events in pregnancies with *both* maternal and fetal heart disease.

**Study design:**

From the CARPREG database, singleton pregnancies (>24 weeks) in patients with structural heart disease that underwent fetal/neonatal echocardiograms were selected and separated in two groups: maternal heart disease only (M-HD) and maternal and fetal heart disease (MF-HD). Differences in adverse fetal/neonatal (death, preterm birth, and small for gestational age) and obstetric (preeclampsia/eclampsia) outcomes between groups were analyzed.

**Results:**

From 1011 pregnancies, 93 had MF-HD. Fetal/neonatal events (38.7% vs 25.3%, p = 0.006) and spontaneous preterm birth (10.8% vs 4.9%, p = 0.021) were more frequent in MF-HD compared to M-HD, with no difference in obstetric events. MF-HD remained as a significant predictor of fetal/neonatal events after adjustment (OR:1.883; 95% CI:1.182–3.000; p = 0.008).

**Conclusions:**

Pregnancies with MF-HD are at risk of adverse fetal/neonatal events and spontaneous preterm birth. Larger studies are needed to determine their association with preeclampsia.

## Introduction

Pregnant patients with heart disease (HD) are at increased risk of adverse fetal/neonatal outcomes [1–3] for a myriad of reasons including maternal genetics, altered maternal hemodynamics [4, 5], cardiac complications during pregnancy [6], and the use of cardiac medications that impact the developing fetus [7]. Placental pathology is also common in this population [8, 9]. Some cardiac diseases, such as coarctation of the aorta [10] or ischemic heart disease, have been associated with hypertensive disorders of pregnancy [11], and other conditions such as the Fontan circulation, are at higher risk of post-partum hemorrhage [1]. Furthermore, patients with congenital heart disease (CHD) are at increased risk of transmission of CHD to their offspring [12–14].

Pregnancies in patients with normal hearts, but complicated by fetal heart disease (FHD) are also at increased risk of adverse fetal events such as preterm delivery and lower birth weight, as well as obstetric complications such as preeclampsia [15–17]. Although this could be attributed to genetic and nutritional factors, placental dysfunction or an “altered maternal-fetal environment“ has been postulated as an important contributor [18–20]. The expression of poor placental function can range from abnormal uterine Doppler interrogation and fetal growth restriction to prematurity [18] and preeclampsia [21].

Large population-based studies have found that pregnancies in patients with structurally normal heart but concomitant FHD are at higher risk of severe maternal morbidity [22], particularly related to the risk of developing preeclampsia [23–25]. Although the exact mechanism of this association is not known, pregnancies complicated with FHD have been found to express lower levels of angiogenic factors like the Placental Growth Factor (PlGF) [15] and higher levels of anti-angiogenic factors such as soluble fms-like tyrosine kinase 1 (sFl-t1) [24]. Furthermore, angiogenic factors, such as the vascular endothelial growth factor receptor (VEGFr) are also thought to be implicated in the formation of the fetal heart [24]. Abnormalities in placental perfusion have been proposed to stem from angiogenic factors imbalance, and this is postulated as one of the mechanisms behind preeclampsia on the maternal side and growth restriction and prematurity on the fetal side [23–26]. This suggests a common pathway between the development of the fetal heart and the formation of the placenta that is the subject of ongoing investigation.

We hypothesized that there may be a “*two-hit*” mechanism whereby pregnancies complicated by *both* maternal and fetal heart disease (MF-HD) would have a higher incidence of adverse fetal/neonatal and obstetric events compared to pregnancies with maternal heart disease (M-HD) only.

## Material and methods

We performed a retrospective analysis of a cohort of pregnant patients with heart disease that were prospectively enrolled between 1995 and 2015 in the Cardiac Diseases in Pregnancy (CARPREG) study [1, 6]. Informed consent was obtained from all subjects at the time of enrollment. Pregnant patients with congenital or acquired structural HD who underwent fetal or neonatal echocardiographic screening for FHD and whose pregnancy progressed beyond 24 weeks of gestational were included in the analysis. Patients with arrhythmias and structurally normal hearts, multiple gestation pregnancies, and patients who refused to participate in the study or with incomplete data were excluded from the final analysis. This study was approved by the local research ethics board, and participants gave informed consent.

### Baseline characteristics

Methods for data collection have been previously described by members of our group [1]. We collected the following variables for each pregnancy: Age, gestational age at first visit, smoking history, comorbidities, and type of maternal heart disease. Markers of disease severity like history of previous cardiac surgery or intervention, New York Heart Association (NYHA) class 3 or 4 or cyanosis, Maternal left ventricular obstruction (LVO) (aortic valve area less than 1.5 cm^2^, peak gradient more than 30 mmHg or mitral valve area less than 2 cm^2^), and cardiac medications used during pregnancy were also collected. All maternal cardiac diagnoses were confirmed by echocardiography at the first antenatal visit. Obstetrical history including gravity, parity, and history of any obstetric complications (hypertensive disorder in a previous pregnancy, history of any of the following: prematurity or premature rupture of membranes, Cesarean delivery, placental insufficiency, fetal growth restriction, fetal death) were also recorded.

### Detection of FHD

As per our clinical protocol, all patients with maternal CHD are referred for fetal and/or neonatal echocardiographic screening, in concordance with current guidelines [27]. Pregnancies complicated by maternal-acquired HD are not routinely referred for fetal and neonatal echocardiogram unless abnormalities are detected in the anatomic scan. The final diagnosis and neonatal outcome were confirmed by a pediatric cardiologist or according to the neonatal medical records and were recorded up to 6 months after delivery.

**Fig. 1:**
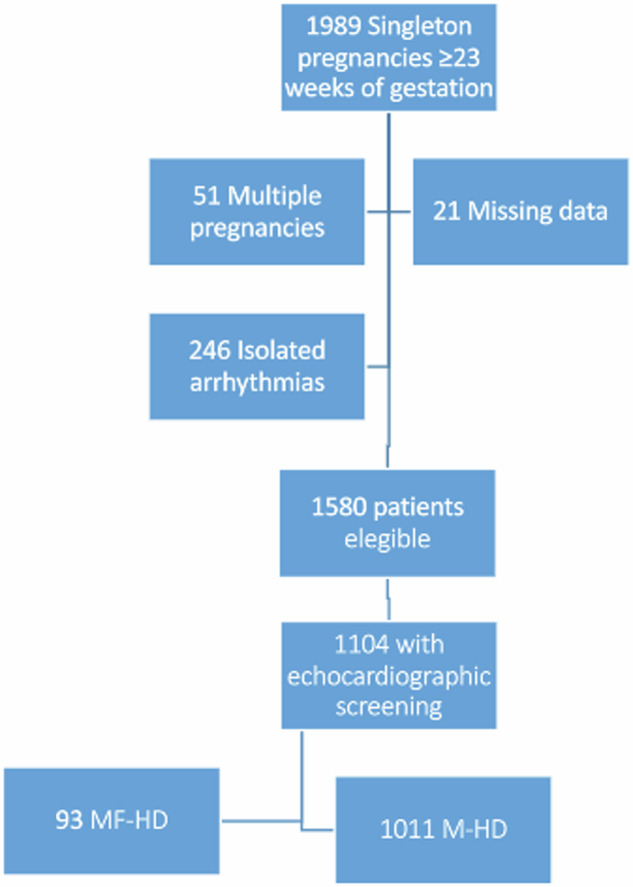
Flow-diagram. M-HD: Maternal heart disease. MF-HD: Maternal and fetal heart disease.

Two groups were assembled: Pregnancies with maternal HD (M-HD) and pregnancies with both maternal and fetal heart disease (MF-HD)*.* Prematurity-related patent ductus arteriosus and neonatal patent foramen ovale were not considered to be FHD and were included in the M-HD only group (6 patients, respectively) (Fig. 1). Maternal and fetal cardiac diagnosis in the MF-HD group were grouped as follows: Left-sided heart disease (LHD), left to right shunt lesions, conotruncal abnormalities, right-sided heart disease (RHD), hypertrophic cardiomyopathy, and Marfan syndrome. Diagnoses not included in the original description were defined as other CHD or other maternal-acquired HD as appropriate.

### Primary endpoints

Primary endpoints were those related to an abnormal maternal-fetal environment. Adverse fetal/neonatal events were defined as any of the following: preterm birth (<37 weeks’ of gestation), extreme prematurity (<32 weeks’ of gestation), fetal death (>24 weeks’ of gestation and before delivery) or neonatal death (Death within the first 28 days of life), and small for gestational age (SGA) (birth weight <10th centile). To further identify the influence of iatrogenic preterm delivery in the primary outcomes, the initiation of labor was stratified according to spontaneous delivery or preterm (<37 weeks’ of gestation) or term induction of labor. Adverse obstetric outcome was defined as diagnosis of preeclampsia and/or eclampsia [28].

### Secondary endpoints

Neonatal secondary endpoints included: birth weight in grams, respiratory distress syndrome (RDS) and/or intraventricular hemorrhage (as per neonatal records), any congenital fetal malformation other than cardiac, and Neonatal Intensive Care Unit (NICU) stay. Obstetric secondary endpoints were post-partum hemorrhage defined as blood loss >500 mL (vaginal delivery) or >1000 mL (Cesarean delivery), which required transfusion or was accompanied by a drop in hemoglobin ≥20 g/L [29], and obstetric death, defined as a death directly related to obstetric complications.

### Data analysis

SPSS statistics version 25.0 for macOS (IBM, Armonk, New York) was used for analysis. Categorical variables are expressed in frequencies and percentages. Continuous variables were tested for distribution type and are presented as mean and standard deviation or median and interquartile range as appropriate. Comparison of baseline characteristics between pregnancies with M-HD and MF-HD was performed using Chi-square, Fisher´s exact test, Students t-test or Mann Whitney U test as appropriate. For the total cohort, a univariable logistic regression model for the identification of predictors of a composite primary outcome (adverse fetal/neonatal events) was done. Spearman’s Rho and Pearson’s were used to test for collinearity when appropriate. There were no highly correlated univariate candidate variables. Univariable predictors with a P value ≤ 0.1, were included in the final multivariable stepwise elimination logistic regression model. A P value < 0.05 (2-sided) was considered significant.

## Results

From 1580 singleton pregnancies that progressed beyond 24 weeks of gestation, 1104 underwent fetal/neonatal CHD screening and were included in the final analysis. 1011 (91.6%) pregnancies had M-HD only, and 93 (8.4%) pregnancies had MF-HD (Fig. 1). Three pregnancies complicated by severe FHD were terminated before the 24th week of gestation and were not included in the study analysis. Baseline characteristics are shown in Table 1. Of the MF-HD group, 77 pregnancies (82.2%) were diagnosed with FHD during prenatal echocardiographic screening, and 16 (17.2%) were diagnosed in the neonatal period. Distribution of HD diagnosis in the MF-HD group is shown in Table 2. Recurrence of CHD in the offspring was noted in 90 pregnancies, with an estimated incidence of 8.7% in the maternal CHD subgroup; similar to prior reports [12–14]. Patterns of recurrence differed across cardiac diagnosis categories. Of note, the MF-HD group had higher frequency of an underlying maternal genetic syndrome, such as Noonan syndrome or Holt Oram than the M-HD only group (9.7% vs 2.4%). The incidence of CHD in the acquired maternal HD subgroup could not be reliably established. History of congenital cardiac fetal abnormalities in previous pregnancies was seen in 9.7% of the MF-HD group. From the entire cohort, 7 patients took Angiotensin Converting Enzyme inhibitor during pregnancy (ACEi). These patients were taking an ACEi prior to conception and had late antenatal assessment during their pregnancy. All ACEi were stopped at the time of the first medical care contact.

**Table 1:** Baseline characteristics.

Baseline characteristics			
	Total (n = 1104)	Maternal HD (n = 1011)	Combined MF-HD (n = 93)
Age	30 ± 5.6	31 ± 5.59	29 ± 6.22
Smoking	83 (7.5)	72 (7.1)	11(11.8)
Medical conditions			
Hypertension	22 (2.0)	22 (2.2)	0 (0)
Diabetes	19 (1.7)	16 (1.6)	3 (3.2)
Thyroid disease	49 (4.4)	48 (4.7)	1 (1.1)
Maternal cardiac disease			
Congenital	1036 (93.8)	949 (93.9)	87 (93.5)
Acquired	68 (6.2)	62 (6.1)	6 (6.5)
Previous cardiac intervention^a^	728 (65.9)	670 (66.3)	58 (62.4)
Previous cardiac surgery	680 (61.6)	626 (61.9)	54 (58.1)
NYHA 3–4 or cyanosis	22 (2.0)	22 (2.2)	0
Maternal left heart obstruction	123 (11.1)	112 (11.1)	11 (11.8)
Cardiac meds during pregnancy			
ACEi	7 (0.6)	6 (0.6)	1 (1.1)
Beta-blockers	175 (15.9)	161 (15.9)	14 (15.1)
Aspirin	73 (6.6)	69 (6.8)	4 (4.3)
Anticoagulation	46 (4.2)	43 (4.3)	3 (3.2)
Nulliparous	617 (55.9)	558 (55.2)	59 (63.4)
Previous hypertensive disorder of pregnancy	24 (2.2)	22 (2.2)	2 (2.2)
Prior obstetrical history^b^	58 (5,3)	55 (5,4)	3 (32)
Congenital cardiac fetal abnormality in prior pregnancies	39 (2.5)	30 (3)	9 (9.7)
Ga at 1st visit – median (IQR)	14 (10–20)	13 (10–20)	14 (11–20.5)

*HD* heart disease, *MF-HD* maternal and fetal heart disease, *NYHA* New York Heart Association functional class, *ACEi* angiotensin converting enzyme inhibitors, *GA* gestational age, *IQR* Interquartile range.

^a^Previous cardiac intervention: surgical or non-surgical.

^b^Prior ob: prematurity, premature rupture of membranes, Cesarean delivery, placental abruption, Fetal growth restriction, Fetal death.

**Table 2:** Frequencies of maternal and fetal/neonatal heart disease underlying diagnosis.

Maternal and fetal heart disease group (n = 93)			
Diagnosis	Mothers n(%)	Diagnosis	Fetuses/Babies n(%)
Left-sided lesions	19 (20)	Left-sided lesions	7 (7.5)
Shunts	32 (34.4)	Shunts	52 (55.9)
Conotruncal defects	11 (11.8)	Conotruncal defects	4 (4.3)
Transposition	3 (3.2)	Transposition	2 (2.1)
Right heart disease	13 (13.9)	Right heart disease	16 (17.2)
Hypertrophic cardiomyopathy	4 (4.3)	Hypertrophic cardiomyopathy	2 (2.1)
Marfan syndrome	6 (6.4)	Others	10 (10.7)
Others	5 (5.3)		

*Left-sided lesions:* Bicuspid aortic valve, aortic stenosis, aortic coarctation, supra valvular or sub valvular aortic membranes, and mitral valve abnormalities.

*Shunts:* atrial septal defect, ventricular septal defect, and atrioventricular septal defect.

*Conotruncal defects:* Double Outlet Right Ventricle, Tetralogy of Fallot, Pulmonary atresia and ventricular septal defect, or truncus arteriosus.

*Transposition:* Levo and dextro transposition of the great arteries.

*Right heart disease:* Ebstein anomaly, pulmonary stenosis, and pulmonary atresia with intact ventricular septum.

*Others:* Tumors, Aneurysm of sinus of Valsalva, coronary fistula, isomerism, complex univentricular heart, etc.

*Acquired:* Valvular regurgitation or stenosis, prior myocardial infarction, etc.

Pregnant patients with MF-HD were younger, with a trend towards higher rates of nulliparity and smoking. Preterm birth occurred in 131 deliveries (11.8%). 60 were spontaneous preterm deliveries, representing 5.4% of the entire cohort. Patients in the MF-HD group had higher rates of spontaneous preterm delivery compared to M-HD only (10.8 vs 4.9%, p = 0.018). There was no difference in the rates of induced preterm delivery between MF-HD and M-HD only (5.4% vs 2.9%, p = 0.199). Five patients in the MF-HD group had preterm induction at 36 weeks’ of gestation due to high-risk maternal cardiac lesions in three patients, and preeclampsia and fetal growth restriction in the remaining two. 3.3% of the cohort underwent either scheduled or emergency preterm Cesarean delivery, with no difference between MF-HD and M-HD groups (6.5 vs 3.0%, p = 0.115). Indications for Cesarean delivery in the MF-HD group were antepartum bleeding in 2 cases, preeclampsia in 1 case, and cardiac indications in 3 cases.

### Primary and secondary endpoints

**Fig. 2:**
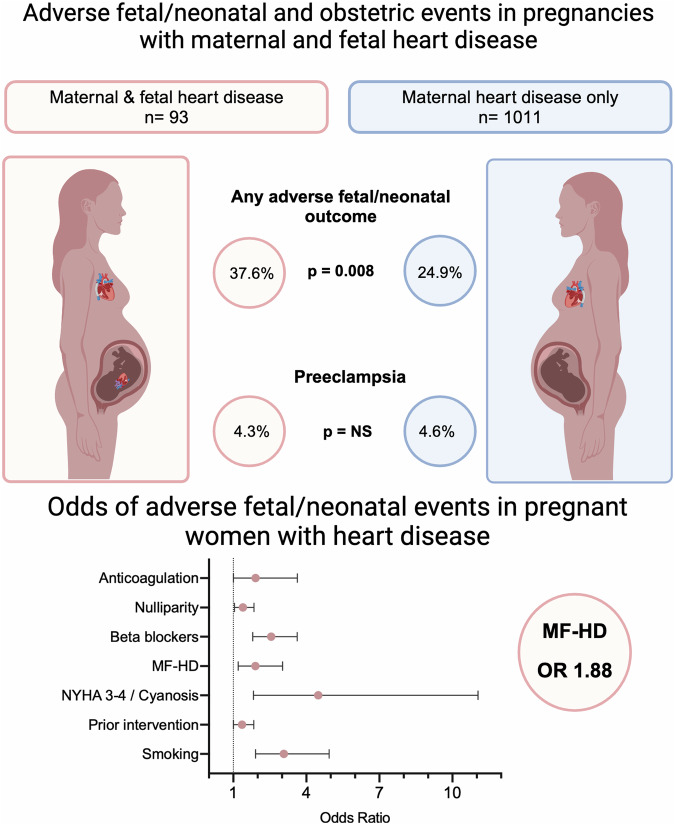
Adverse Fetal/Neonatal and obstetric events in pregnancies with maternal and fetal heart disease. Variables included in the analysis: MF-HD Maternal and fetal heart disease, active smoking, History of previous interventions, New York Heart Association class 3 or 4 or cyanosis. Maternal left ventricular obstruction: Aortic valve area less than 1.5 cm^2^, peak gradient more than 30 mmHg or mitral valve area less than 2 cm^2^. Diabetes prior to pregnancy; Use of beta-blockers during pregnancy; Anticoagulation during pregnancy (Coumadin or heparin); Maternal age >18 or <35 years.

Analysis of primary and secondary endpoints is shown in Table 3 and Fig. 2. Overall, the incidence of adverse fetal/neonatal events was 26%. Adverse fetal/neonatal events were more common in the MF-HD group than in M-HD (36.7 vs 24.6%, p = 0.008). Individual analysis showed a higher incidence of prematurity (22.6 vs 10.9%, p = 0.001) in the MF-HD group than in the M-HD group. There was no statistical difference in the rates of extreme prematurity, fetal or neonatal death, or preeclampsia between MF-HD and M-HD groups, respectively. Regarding the secondary endpoints, newborns in the MF-HD group had higher rates of respiratory distress syndrome other congenital fetal abnormalities, and NICU stay, than newborns in the M-HD group. There was no difference in the incidence of obstetric death or post-partum hemorrhage across groups.

**Table 3:** Primary and secondary endpoints.

Primary and secondary endpoints				
	Total (n = 1104)	Maternal HD (n = 1011)	Combined MF-HD (n = 93)	P
Primary endpoints: adverse fetal and neonatal events				
Fetal events (total)	287 (26)	252 (24.9)	35 (37.6)	**0.008**
Fetal or neonatal death (up to 28 days)	5 (0.5)	4 (0.4)	1 (1.1)	0.370
Preterm birth (<37 weeks’)	131 (11.9)	110 (10.9)	21 (22.6)	**0.001**
Extreme preterm birth (<32 weeks’)	22 (2.0)	20 (2.0)	2 (2.2)	0.909
Gestational age at delivery^a^	39 (38–40)	39 (38–40)	38 (37.5–40)	0.220
SGA (less than 10th centile)	180 (16.3)	162 (16)	18 (19.4)	0.406
Primary endpoint: obstetric events				
Preeclampsia	51 (4.6)	47 (4.6)	4 (4.3)	0.879
Secondary endpoints: adverse fetal and neonatal events				
Birth weights^a^	3200 (2840–3546)	3200 (2855–3545)	3200 (2600–3550)	0.205
RDS	33 (3.0)	27 (2.7)	6 (6.5)	**0.047**
IVH	3 (0.5)	3 (0.5)	0	1.000
Other congenital fetal abnormalities^b^	32 (2.9)	25 (2.5)	7 (7.5)	**0.008**
NICU	160 (14.5)	130 (12.9)	30 (32.3)	**<0.001**
Secondary endpoints: obstetric events				
Obstetric death	0			1.000
Post-partum hemorrhage	47 (4.3)	47 (4.6)	0 (0.0)	0.997

*RDS* respiratory distress syndrome, *IVH* intraventricular hemorrhage, *SGA* small for gestational age, *NICU* neonatal intensive care unit stay. P-values (bold indicate significance <0.05).

^a^Median (Interquartile range 25–75).

^b^Congenital non-cardiac fetal abnormalities.

### Multivariable analysis

Predictors of adverse fetal/neonatal events were identified in the univariate analysis (Table 4). Other clinically relevant variables and predictors of adverse maternal cardiac events were also identified and included in the multivariable logistic regression analysis to account for the severity of maternal heart disease (Table 5). The presence of MF-HD remained as a significant predictor of adverse fetal/neonatal events (odds ratio (OR): 1.883; 95% confidence interval (CI): 1.182–3.000; p = 0.008) in the multivariable analysis. In a secondary analysis, MF-HD remained as the only significant predictor of spontaneous preterm delivery (p = 0.026. OR 2.301, 95% CI 1.105–4.791) after adjusting for the same clinically and statistically relevant variables.

**Table 4:** Univariate predictors of adverse fetal/neonatal events.

Univariate predictors of adverse fetal/neonatal events			
	No adverse fetal/neonatal events (n = 817)	Adverse fetal/neonatal events (n = 287)	P value^b^
Maternal and fetal heart disease	58 (7,1)	35 (12.2)	**0.008**
Age <20 or >35	158 (19.5)	60 (20.5)	0.688
Smoking	40 (4.9)	43 (15)	**<0.001**
Medical conditions			
Hypertension	13 (1.6)	9 (3.1)	0.114
Diabetes	10 (1.2)	9 (3.1)	**0.039**
Thyroid disease	32 (3.9)	17 (5.9)	0.159
Maternal cardiac disease			
Congenital	778 (95.2)	258 (89.9)	**0.002**
Acquired	39 (4,8)	29 (10,1)	
Previous Cardiac Intervention^a^	522 (64.3)	203 (70.7)	**0.047**
NYHA 3–4 or cyanosis	9 (1,1)	13 (4,5)	**0.001**
Maternal left heart obstruction	83 (10.2)	40 (13.9)	0.081
Cardiac Meds during pregnancy			
ACE inhibitors	4 (0.5)	3 (1.0)	0.319
Beta-blockers	98 (12.0)	77 (26.8)	**<0.001**
ASA	48 (5.9)	25 (8.7)	0.098
Anticoagulation	23 (2.8)	23 (8,0)	**<0.001**
Hx of HTN disorder (Previous preg)	19 (2.3)	5 (1.7)	0.561
Nulliparous	442 (54,1)	175 (61)	**0.044**
Prior Obstetrical history^b^	38 (4.7)	20 (67,0)	0.133
Congenital cardiac fetal abnormality in prior pregnancies	33 (4.0)	6 (2.1)	0.131
GA at 1st visit, Median (IQR)	14 (10–20)	13 (10–20)	0.737

*HD* Heart Disease, *MF-HD* Maternal and Fetal Heart Disease, *NYHA* New York Heart Association functional class, *ACEi* Angiotensin Converting Enzyme Inhibitors, *GA* Gestational age. P-values (bold indicate significance <0.05).

^a^Previous cardiac intervention: surgical, percutaneous or arrhythmia intervention.

^b^Prior obstetric history: prematurity, premature rupture of membranes, cesarean delivery, placental insufficiency, fetal growth restriction, fetal death.

**Table 5:** Multivariable analysis for predictors of adverse fetal/neonatal events.

Multivariable analysis for predictors of adverse fetal/neonatal events			
	OR	CI	P
MF-HD	1.883	1.182–3.000	0.008
Active smoking	3.261	2026–5250	<0.001
Previous intervention	1.361	1.000–1.850	0.050
Beta-blockers	2.641	1.859–3.751	<0.001
Nulliparity	1.504	1.111–2035	0.008
Anticoagulation	1.965	1.033–3.736	0.039
Prior obstetric risk factor	2047	1.111–3.774	**0.022**
NYHA 3-4 or cyanosis	4.334	1.760–10,671	<0.001

Variables included in the analysis: MF-HD = maternal and fetal heart disease; smoking history; maternal left heart obstruction; previous history of hypertension; previous history of diabetes; use of beta-blockers during pregnancy; use of anticoagulation during pregnancy; maternal age more than 35 or less than 20, and prior obstetric risk factors: prematurity, premature rupture of membranes, cesarean delivery, placental abruption, fetal growth restriction or fetal death. P-values (bold indicate significance <0.05).

## Discussion

Our study shows that, when compared to pregnancies with M-HD only, pregnancies with both MF-HD are at increased risk adverse fetal/neonatal events and spontaneous preterm birth, suggesting that there is a complex association between both maternal and fetal cardiovascular status and the placenta, and supporting a *two-hit* hypothesis. However, while maternal-placental syndrome has been reported to be increased in pregnancies in women with underlying heart disease [3] and in pregnancies with isolated FHD [25], we did not find any difference in the rates of preeclampsia in pregnancies with MF-HD when compared to M-HD only.

It is known that pregnancies complicated by simple and complex forms of fetal heart disease are at increased risk of adverse fetal/neonatal events such as preterm birth, low birth weight, and SGA. A population-based study analyzing 6863 infants born with critical forms of CHD, found that infants with critical forms of CHD were more commonly SGA when compared to newborns without CHD (16.3 vs 8.1%, P < 0.0001) [20]. Furthermore, a systematic review on newborns with isolated CHD showed a higher risk of SGA infants irrespective of the type or severity of the CHD [30]. The prevalence of SGA In our study was higher than in the Canadian population (16% vs 8%, respectively) [31], although there was no difference between MF-HD and M-HD only groups. This could explained by various maternal factors, such as low cardiac output or the use of medications such as beta-blockers during pregnancy, which may affect fetal growth [2, 7]. However, our findings may also be reflecting the small numbers in the MF-HD group and the low prevalence of critical forms of CHD in the cohort.

The incidence of preterm birth in pregnancies with *both*, maternal and fetal heart disease, had not been explored before. Prior studies have documented a 13.5% incidence of preterm delivery in fetuses born with critical forms of congenital heart disease and an increased risk of preterm delivery when compared to the general population [32]. The incidence of preterm birth has also been shown to be increased in pregnancies with M-HD [3]. The overall incidence of preterm birth in our study was higher than the estimated for singleton pregnancies in Canada [33] (11.9 vs 6.2%, respectively) and was significantly increased in the MF-HD group (22.6% vs 10.9%, p = 0.001) compared to the M-HD only. Given that the time at induction of labor or scheduled Cesarean delivery did not differ between groups, there is a low likelihood of iatrogenic preterm birth bias in our cohort. Furthermore, MF-HD remained as a significant predictor of adverse fetal/neonatal events and spontaneous preterm birth in the multivariable analysis after adjustment for other several known risk factors for maternal cardiac events in pregnancy and adverse neonatal outcomes, highlighting the multi-factorial nature of the fetal/neonatal complications.

Although SGA and prematurity are common in pregnancies with FHD, studies have also shown an increased risk of severe maternal morbidity and preeclampsia development due to an imbalance between pro-angiogenic and anti-angiogenic factors [25] However, we did not find this association in pregnancies with both MF-HD. The incidence of preeclampsia in the MF-HD group and the M-HD group were similar and did not differ from those seen in the general population [34]. There are several potential explanations to these findings like the difference in preeclampsia rates across various types of maternal HD [10, 11, 35] or the frequent use of aspirin and or beta-blockers in cardiac populations, which could potentially mitigate the risk for developing preeclampsia. Finally, the risk of developing preeclampsia and its severity may increase as a pregnancy approaches to term, hence the incidence of preeclampsia in the MF-HD may be blunted due to a high incidence of prematurity.

Women with structural heart disease are prone to several factors that can affect the maternal-fetal placental axis, predisposing to poor placental function. For instance, patients who have had a prior intervention are often exposed to sensitization secondary to the use of homograft’s and blood products transfusions; placentas from patients with CHD have been found to express villitis of unknown etiology, suggesting an underlying immunological reaction [17]. A reduction in the cardiac output due to an abnormal systemic ventricular function, or the use of some cardiac medications could also predispose to placental ischemia [8]. Furthermore, conditions with increased systemic venous pressure have also been linked to abnormal uterine

Doppler interrogation [36] and histopathological placental abnormalities [37]. Findings of our study suggest further alteration in the maternal-placental-fetal axis in pregnancies with both, maternal and fetal heart disease, leading to more adverse fetal/neonatal events and supporting a two-hit theory.

Specialized obstetric centers caring for women with congenital or acquired HD should be aware of the risk of CHD transmission and increasing risk of fetal/neonatal complications. This could be achieved by establishing pathways to detect concomitant FHD early in pregnancy and care-plans focused in a close maternal-fetal surveillance and interventions to allow for a term delivery to improve neonatal outcomes.

### Areas of future research

Analyzing values of vascular growth factors such as PLGF of SFl-t1 [23, 38] and its association with clinical outcomes and placental pathology could give more insight into the etiology of adverse fetal/neonatal outcomes in this population and its relationship with preeclampsia. Further studies that analyze the impact of FHD in pregnancies complicated by maternal-acquired HD and preeclampsia risk in this population are needed. Underlying mechanisms that could explain the association between maternal HD and the incidence of adverse fetal events is also an interesting topic for future studies.

### Strengths and limitations

The CARPREG study is a multi-center registry that focused on pregnancies complicated by maternal HD. As a routine practice, most of the patients are sent to prenatal screening of FHD, permitting a comprehensive assessment of pregnancies with both conditions and allowing us to evaluate the influence of FHD in the outcomes. Although the number of pregnancies affected by both, maternal and fetal HD is small, this was a study conducted in one of the largest cohorts worldwide. However, due to a reduced number of fetal HD screening in women with acquired HD, results from our study cannot be extrapolated to this specific group of patients. Newer preeclampsia biomarker-screening modalities are not routinely measured in our clinical practice, and placentas are not routinely sent to pathology, which limits our ability to link our results to potential pathophysiological mechanisms. Lastly, the pregnancy and heart disease clinic mostly follows pregnant women with heart disease and, therefore, we lack of a group of pregnancies with FHD only to serve as control.

## Conclusions

Pregnancies with MF-HD are at increased risk of adverse fetal/neonatal events when compared to pregnancies with M-HD only. Pregnancies where maternal and fetal heart disease co-exists benefit from close surveillance and interventions to allow for a term delivery to improve neonatal outcomes. Further studies are needed to elucidate associations between MF-HD and the risk of developing preeclampsia.

## Data Availability

Information will be promptly available to referees and to readers upon request.
